# Photoactivated release of membrane impermeant sulfonates inside cells†

**DOI:** 10.1039/d0cc07713e

**Published:** 2021-03-12

**Authors:** Stuart T. Caldwell, Sean N. O'Byrne, Calum Wilson, Filip Cvetko, Michael P. Murphy, John G. McCarron, Richard C. Hartley

**Affiliations:** School of Chemistry, University of Glasgow Glasgow G12 8QQ UK richard.hartley@glasgow.ac.uk; Strathclyde Institute of Pharmacy and Biomedical Sciences, University of Strathclyde, 161 Cathedral Street Glasgow G4 0RE UK; MRC Mitochondrial Biology Unit, Hills Road, University of Cambridge CB2 0XY UK

## Abstract

Photouncaging delivers compounds with high spatial and temporal control to induce or inhibit biological processes but the released compounds may diffuse out. We here demonstrate that sulfonate anions can be photocaged so that a membrane impermeable compound can enter cells, be uncaged by photoirradiation and trapped within the cell.

## Abstract 2

Photocaged sulfonate delivers membrane impermeant compounds to cells.

## Graphical abstract

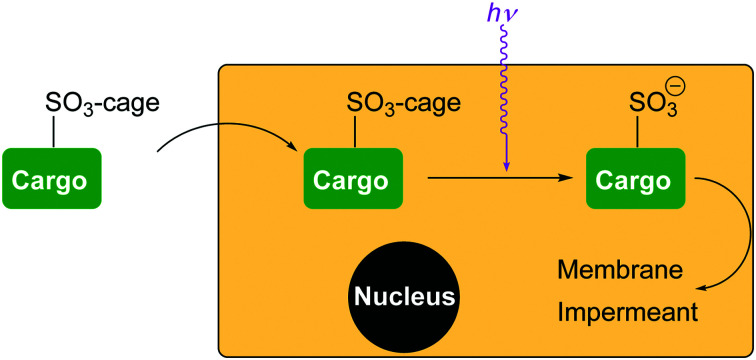

The delivery of drugs or probes to cells often relies on diffusion into the cell. Under these circumstances, the compound can subsequently freely diffuse out of the cell in the same way. Therefore, some chemical change is necessary to retain it. One approach is to protect the compound in a way that is susceptible to endogenous processes in the cell, such as enzyme-catalysed hydrolysis, and for the released compound to become membrane impermeant as a result of increased charge. This is successful for many sensors. For example, BAPTA-based calcium sensors can be caged as neutral acetoxymethyl (AM) esters,^1^ and when released the concentrated charge of four carboxylate anions prevents the sensor diffusing out of the cell again. In a similar way, esters have been combined with photocaged fluorophores^2–4^ and bioactive compounds^5,6^ (Fig. 1 for examples). Esterase release of the charged groups result in the photocaged compounds being trapped inside cells. Photouncaging using UV light generates the switched-on fluorophore or bioactive still trapped inside the cell. We believed a more general method would be to cage the ion of a strong acid incorporated into the structure of the compound that is to be retained in the cell. We now report that the delivery of membrane impermeant sulfonate compounds to cells followed by photochemical trapping is possible (Fig. 2).

**Fig. 1:**
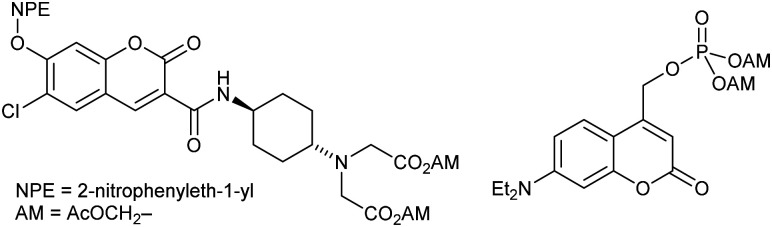
Examples of doubly caged compounds. Doubly caged coumarin (ref. 3) and doubly caged phosphate (ref. 6).

**Fig. 2:**
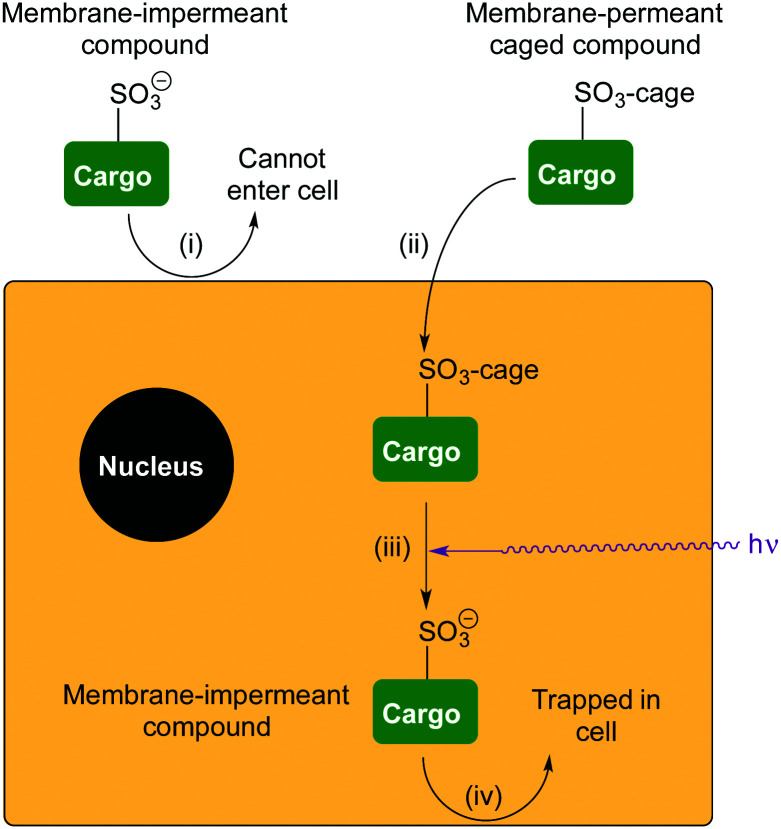
Delivery and retention of a membrane-impermeant compound in cells. (i) A compound is membrane-impermeant and cannot diffuse into the cell because it includes a sulfonate anion; (ii) the compound is caged as a neutral sulfonate ester, which can diffuse into the cell; (iii) photo-uncaging releases the membrane-impermeant sulfonate anion; (iv) compound cannot diffuse out of the cell again.

Photouncaging of aryl sulfonates using short wavelength UV light of typically <330 nm^7^ has been used for the generation of acids or the deprotection of sulfonates.^8^ 2-Nitrobenzyl sulfonate esters^9^ such as “caged sulfate”^10^ have also been used as photoacids, while irradiation of 1-(2-nitrophenyl)-ethylhexadecyl sulfonate^11^ has been used *in vivo* for intracellular acidification, but caging in this way has never been suggested for the delivery of membrane-impermeant compounds to cells. This may be because benzylic sulfonates are easily hydrolysed and are reactive alkylating agents. The Miller group^12,13^ has demonstrated that sulfonate anions can be caged as α-trifluoromethylbenzylic sulfonate esters, which are resistant to hydrolysis by *S*_N_1 or *S*_N_2. We reasoned that incorporation of an α-trifluoromethyl group would ensure stability of an *ortho*-nitrobenzylic photocage prior to activation and may increase the efficiency of uncaging.^14^ We chose to use a sulfonated dye as a proof of principle to demonstrate that the α-trifluoromethyl-*ortho*-nitrobenzyl (TFNB) group would allow membrane-impermeant sulfonates to enter cells and be retained upon uncaging. The use of a fluorophore allows visualization of uptake and also allows assessment of the effect of sulfonation on membrane permeation. To demonstrate the principle we chose sulforhodamine B **1** because it is one of the most widely utilized fluorescent dyes and is known to be cell impermeable.^15,16^ We proposed that bis-caged sulforhodamine (BCSR) **2** would be membrane permeant. Photouncaging would then lead to sulforhodamine B **1** being trapped within the cell, demonstrating the utility of TFNB for delivery of sulfonates (Scheme 1). (See ESI,† for synthesis).

**Scheme 1:**
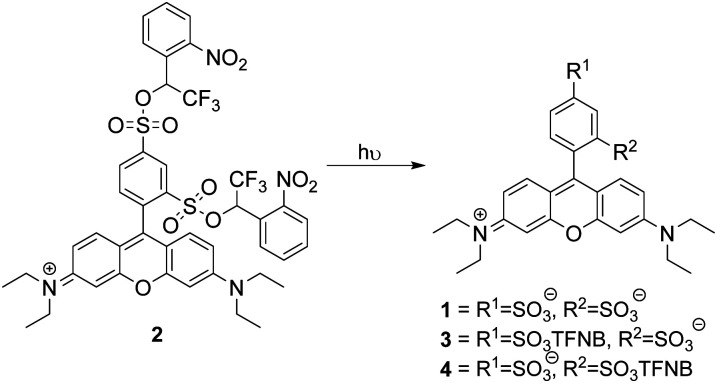
Sulforhodamine bis-TFNB ester **2** and potential products from photochemical uncaging. TFNB = α-trifluoro-*ortho*-nitrobenzyl.

We first monitored the uncaging using fluorescence. Fluorescence emission of BCSR **2** in pH 7.4 buffer was almost completely quenched in buffer due to photoinduced electron transfer (PeT) quenching by the nitroarene groups. This increased 118-fold upon irradiation at 365 nm (330 W m^−2^) for 90 min (Fig. 3).

**Fig. 3:**
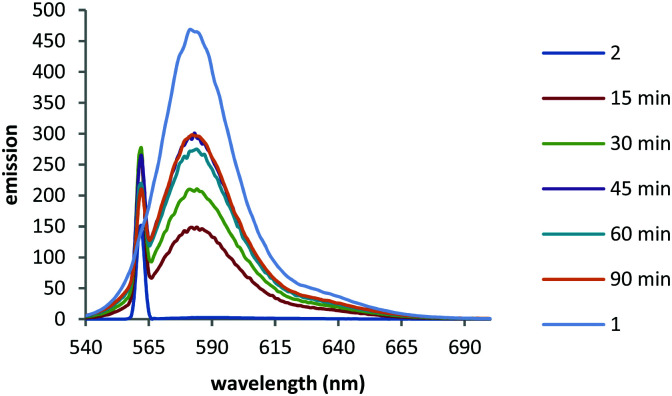
Irradiation of a 10 μM solution of caged TFNB diester **2** in PBS buffer (pH 7.4) using UV (365 nm) light over time. 1 = sulforhodamine sodium salt 10 μM in PBS buffer (pH 7.4). Excitation 560 nm.

LCMS analysis of the uncaging revealed a mixture of products due to incomplete uncaging and some partial deethylation of the amines.^17–19^ Within 15 minutes only 1% of the starting material remained with 24% **1**, 50% **3** and 25% **4** (including products from loss of the ethyl group) observed (Scheme 1), showing that removal of the first caging group was rapid and this was followed by a slower second uncaging. Integration of the LC chromatogram showed that 70% full uncaging had occurred within 60 min irradiation, the remainder being a mixture of monouncaged products **3** and **4** (Fig. S1B and A, ESI,† for assignment of LCMS peaks). BSCR **2** was relatively stable in pH 7.4 buffer remaining 85% unchanged after 4 days at 37 °C in the dark (Fig. S1D, ESI†). On the other hand, *ortho*-nitrobenzyl derivatives lacking the α-trifluoro group were unstable.

Next, we used BSCR **2** to show that the TFNB group allowed uptake, release and retention of membrane impermeant sulfonates. BCSR **2** was taken up by HeLa cells. Excitation at 488 nm revealed a pattern of fluorescence for BCSR **2** that was colocalised with the mitochondrial dye, MitoTracker green, (Fig. 4). This confirmed that caging the sulfonates with TFNB allowed an otherwise membrane-impermeant compound to enter cells. Many rhodamine-based dyes accumulate several-hundred-fold in the mitochondrial matrix^20^ because they are lipophilic cations and there is a 120–160 mV membrane potential across the mitochondrial inner membrane, which is negative inside. Therefore, we compared the behaviour of BCSR **2** against another rhodamine dye, TMRE, as a control. The control compound colocalised with MitoTracker green, and as expected this colocalisation was lost when the membrane potential was removed by FCCP. On the other hand, colocalisation of BCSR **2** and MitoTracker green was unchanged by the addition of FCCP. This demonstrates that the colocalisation of BSCR **2** with mitochondria is not the result of membrane-potential driven uptake into the matrix.

**Fig. 4:**
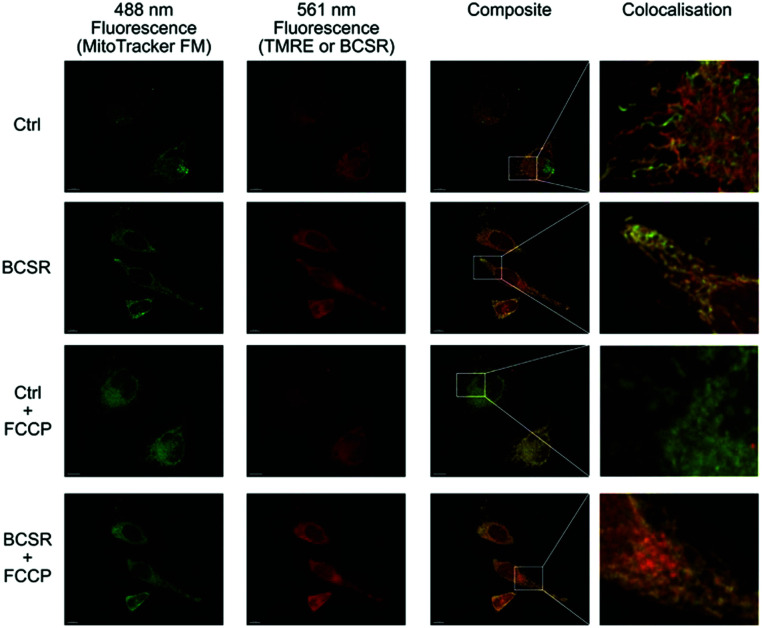
Association of BSCR **2** with mitochondria is not membrane potential dependent. 3D maximum projection images showing fluorescence obtained with HeLa cells incubated with 100 nM MitoTracker green FM (488 nm excitation, first column) and either 1 μM Tetramethylrhodamine ethyl ester control (TMRE, Ctrl) or caged TFNB sulfonate diester **2** (561 nm excitation second column), composite merge of the two fluorescent channels (third column) and an enlargement of regions to show colocalisation. Both BSCR **2** and TMRE show similar patterns of colocalisation with MitoTracker green FM (top two rows). The addition of FCCP (10 μM) eliminates the mitochondrial membrane potential and TMRE staining, but does not have an observable effect on MitoTracker green FM or the caged TFNB sulfonate diester **2**, with its colocalisation staying intact (row three and four, respectively). All scale bars = 10 μm.

Uptake and colocalisation with MitoTracker green was also observed when HEK 293T cells (Fig. S2, ESI†) were incubated with BCSR **2**. Localised irradiation of mitochondria at 355 nm using a laser gave no increase in fluorescence but instead showed a loss of fluorescence. However, surprisingly, wide field excitation at 555 nm (rhodamine excitation wavelength used for imaging) for 5 min led to an approximate 2-fold increase in fluorescence emission and this increase in emission remained within the cell (Fig. 5A and B and Videos 1, 2, ESI†). Cells that were not irradiated with 555 nm light showed negligible increase in fluorescence over the same time period (Fig. 5C and Videos 3, 4, ESI†). Therefore, the observed increase in fluorescence was spatially controlled by visible light and is consistent with photouncaging and is not *via* an alternative hydrolysis mechanism. Notably, the fluorescence increased throughout the cell, indicating redistribution of the dye, consistent with the decrease in fluorescence upon localised irradiation of mitochondria.

**Fig. 5:**
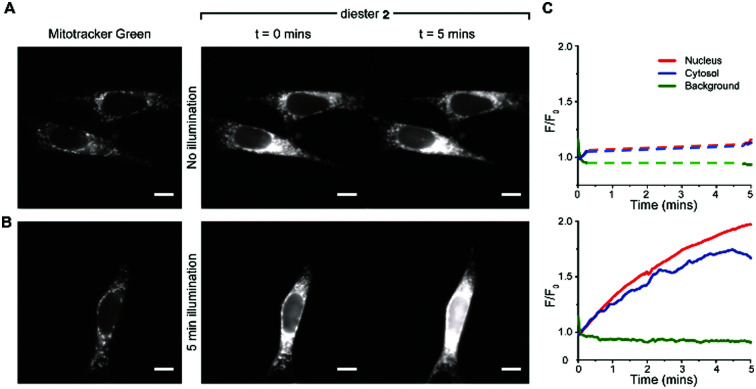
BSCR **2** uncages in visible light. (A) *left-panel* representative images showing the fluorescence signal obtained with 488 nm excitation of mitotracker green (100 nM; left panel). The right panel shows fluorescence signals obtained with 555 nm excitation of diester **2** at the initial exposure (0 min) and at 5 min. There was no light exposure between the two recording periods at 0 min and 5 min. (B) Left panel again shows a mitotracker green image (488 nm excitation) of the mitochondria. The *right-panel* shows the fluorescence images obtained with a continuous 5 minutes exposure to 555 nm excitation of diester **2**. The images shown were taken at commencement and conclusion of a five minutes period. (C) Background-corrected fluorescence intensity (*F*/*F*_0_) traces plotted for the cells shown in A and B. The top panel shows the fluorescence signal obtained from cells that were illuminated only for the 10 seconds at the beginning and the end of the five minutes recording period. During this time (without illumination), the fluorescence signal did not increase. The bottom panel shows the fluorescent signals obtained from cells that were illuminated throughout the recording period. With constant illumination, the fluorescence signal arising from diester **2** increased with time. All scale bars = 10 μm.

The observed redistribution within the cell upon uncaging (Fig. 5 and Videos 1–4, ESI†) is consistent with BCSR **2** associating with the outer surface of the mitochondrial membrane and the highly water-soluble sulforhodamine **1** dissociating upon uncaging. To confirm that this redistribution was due to full uncaging to sulforhodamine B **1**, we confirmed that the monocaged compound **3** showed a similar localisation pattern to BCSR **2** (Fig. S3, ESI†).

Photouncaging of 2-nitrobenzyl groups is typically achieved using UV light^21^ but substituted nitrobenzyl groups have been uncaged at 420 nm^22^ so the increase in fluorescence observed when irradiating TFNB sulfonate diester **2** in cells with 555 nm light was unexpected. We therefore wished to confirm the photouncaging *in vitro*. The UV/Vis absorption spectrum of TFNB sulfonate diester **2** in buffer showed a maximum absorption at ∼586 nm (Fig. S4, ESI†). We therefore irradiated a 10 μM degassed solutions of sulfonate diester **2** at 595 nm (∼57 W m^−2^) with *a* ±5 nm bandpass filter in pH 7.4 buffer and monitored the reaction by LCMS and fluorescence. BCSR **2** was almost completely converted to a mixture of sulfonate monoesters **3** and **4** and sulforhodamine B **1** within 30 min (plus compounds arising from partial deethylation of the amines) giving a 7 : 16 : 1 ratio of **1 **: (**3 + 4**) : **2** (Fig. S1C, ESI†). After 90 min irradiation at 595 nm 62% of the dye was fully uncaged and showed a 170 fold increase in fluorescence emission (Fig. S5, ESI†). The greater increase in emission intensity upon uncaging with 595 nm light compared to 365 nm light is presumably due to less photobleaching when using low energy long wavelength light. Thus, the increase in fluorescence when cells treated with diester **2** are irradiated with 555 nm light is congruent with photouncaging and formation of sulforhodamine B **1**. The observed fluorescence increase when BCSR **2** is irradiated with visible light is due to photouncaging. However, this cannot proceed *via* the usual mechanism of uncaging of 2-nitrobenzyl groups since there is insufficient energy to form the intermediate aci–nitro^23^ complex. We therefore propose that the unexpected uncaging using visible light (555–595 nm) proceeds *via* a *S*_RN_1 mechanism.^24–27^ PeT^28^ from the excited state rhodamine to the nitrobenzyl group results in an aryl radical anion which ultimately cleaves to give the uncaged sulfonate. The uncaging of a picolinium salts has been reported to proceed *via* a similar mechanism.^29–31^

Taken together, these results confirmed that TFNB can be used to cage a membrane impermeant sulfonate allowing it to enter the cells and be photouncaged within cells to release the membrane impermeant sulfonate, which is retained (Fig. 4, 5 and Videos, ESI†). TFNB can be removed in the usual way with UV light (Fig. 3 and Fig. S1B, ESI†), opening the way for the usual spatiotemporal control,^21^ and in the specific case of the dye used to illustrate delivery, it could also be removed with visible light (Fig. S1C and S5, ESI†) to give release only in the irradiated cells (Fig. 5 and Videos, ESI†).

In summary, we have demonstrated for the first time that sulfonic acids can be successfully photocaged as their TFNB sulfonates to deliver membrane-impermeant compounds to cells. The TFNB sulfonate diester of the membrane impermeant dye, sulforhodamine B **1**, can enter cells and can there be uncaged not only by UV light, but also by yellow light. This unusual uncaging at a long wavelength is believed to proceed *via* photoinduced electron transfer from the rhodamine to the TFNB cage. The ability to trap compounds within cells by generating a salt of a strong acid *in situ* using light without the need for endogenous enzymes or nucleophiles allows for the delivery of drug and probes that can be retained near their area of action without diffusing out of the cell. Coupled with the spatial and temporal control typically endowed by photoactivation,^21^ this new method of delivery will be of great use.

This work was supported by Wellcome Trust awards to RCH (092292/B/10/Z and 110158/Z/15/Z), JGM (202924/Z/16/Z), CW (204682/Z/16/Z), MPM (110159/A/15/Z) and FC (PhD programme in Metabolic and Cardiovascular Diseases, RG88195), and by the Medical Research Council UK (MC_U105663142) for MPM.

## Conflicts of interest

There are no conflicts to declare.

## Supplementary Material

CC-057-D0CC07713E-s001

CC-057-D0CC07713E-s002

CC-057-D0CC07713E-s003

CC-057-D0CC07713E-s004

CC-057-D0CC07713E-s005
