# Supplementary material for: Photoactivated release of membrane impermeant sulfonates inside cells
Source: Chem Commun (Camb). 2021 Mar 12;57(32):3917–20. doi: 10.1039/d0cc07713e (PMC7611313; doi:10.1039/d0cc07713e)
Supplement: CC-057-D0CC07713E-s001 [file CC-057-D0CC07713E-s001.pdf]

Electronic Supplementary Material (ESI) for Chemical Communications.
This journal is © The Royal Society of Chemistry 2021

Electronic supplementary information for Photoactivated release of membrane impermeant sulfonates inside cells.

Stuart T. Caldwell,[a] Sean N. O'Byrne,[a] Calum Wilson,[b] Filip Cvetko,[c] Michael P. Murphy,[c] John G. McCarron[b] and Richard C. Hartley*[a]

[a]School of Chemistry, University of Glasgow, Glasgow G12 8QQ, U.K.

[b]Strathclyde Institute of Pharmacy and Biomedical Sciences, University of Strathclyde, 161 Cathedral Street, Glasgow G4 0RE, U.K.

[c]MRC Mitochondrial Biology Unit, Hills Road, Cambridge CB2 0XY, U.K.

Table of Contents

Author Contributions S2

General information on chemical synthesis S2

Synthesis of BCSR **2** S2

Synthesis of moncaged sulforhodamine **3** S3

General method for photouncaging S4

Cell culture and imaging conditions S4

Figure S1
A - LCMS traces after the irradiation of BCSR **2** at 365 nm light after 30 min and structural assignment of peaks based on m/z data.
B - LC-UV traces after the irradiation of BCSR **2** at 365 nm light over time
C - LC-UV traces after the irradiation of BCSR **2** at 595 nm light over time
D - LC trace showing stability of BCSR **2** after 4 days S5

Figure S2 - BCSR **2** colocalises with mitochondria in HEK293T cells. S6

Figure S3 - Cell uptake and localization of monocaged sulforhodamine **3** S7

Figure S4 - UV/Vis absorption spectra of BCSR **2**. S7

Figure S5 - Fluorescence increase after the irradiation of BCSR **2** at 595 nm light over time. S7

Video Legends S8

^{1}H, ^{13}C and ^{19}F NMR spectra of BCSR **2**. S9-S11

^{1}H, ^{13}C and ^{19}F NMR spectra of **3**. S12-S13

Author Contributions

The study was conceived by RCH, JGMcC and MPM. Chemical synthesis and in vitro experiments were carried out by STC and SO'B under the supervision of RCH at the University of Glasgow. Uptake and localisation experiments in HeLA cells were caried out by FC under the supervision of MPM at the MRC Mitochondrial Biology Unit. Uptake, localisation and uncaging experiments in HEK293T were undertaken by CW under the supervision of JGMcC at the University of Strathclyde. RCH coordinated the manuscript and all authors contributed to its preparation.

General information on chemical synthesis

Reagents were obtained from commercial suppliers and used without further purification. Acetonitrile and dichloromethane were dried using a Puresolv solvent. The reaction under an inert atmosphere was carried out using oven-dried glassware and solvents were added *via* syringe. ^{1}H and ^{13}C spectra were obtained on a Bruker AVIII spectrometer operating at 400 or 101 MHz, respectively or a Bruker AVIII operating at 500 and 126 MHz respectively. All coupling constants were measured in Hertz. Deuterated solvents contained trimethylsilane (TMS) as a reference compound. DEPT was used to assign the signals in ^{13}C NMR spectra as C, CH, CH_2 and CH_3. In the interpretation of the ^{1}H NMR spectrum of the 1:1 diastereomeric mixture, where signals overlap, we write $1H^{A\ and\ B}$ to indicate that the integration corresponds to 1H from diastereomer A and 1H from diastereomer B. Where the signals are not coincident, we write $1H^{A\ or\ B}$ to indicate that the integration corresponds to 1H from either diastereomer A or diastereomer B but not both. Mass spectra (MS) were recorded on a Jeol JMS700 (MStation) spectrometer for EI and CI or Bruker Microtof-q for ESI. Purification was conducted using a Biotage Isolera automated system, SpectraSystem P2000 or a Shimadzu Prominence HPLC system

Synthesis of BCSR 2

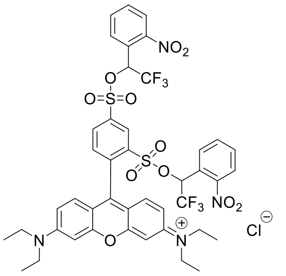

Phosphorus oxychloride (50 µl, 0.51 mmol, 6.0 eq) was added to a suspension of sulforhodamine (50 mg, 0.086 mmol) in MeCN (1 ml) and the suspension heated to 60°C under an atmosphere of argon overnight. After cooling to RT the resulting solution was concentrated under vacuum, the residue dissolved in dichloromethane (1 ml) and 1-(o-nitrophenyl)-2,2,2-trifluoroethanol (76 mg, 0.344 mmol, 4.0 eq), DMAP (~1 mg) and Hunigs base (88 µl, 0.516 mmol, 6.0 eq) added. The solution was stirred at RT for 2h then diluted with dichloromethane (5 ml), washed with 1M hydrochloric acid (2 x 10 ml), dried over sodium sulfate and concentrated under vacuum. The residue was purified by column chromatography using a 10 g SNAP ultra cartridge eluting dichloromethane increasing to 8% methanol. The product containing fractions were further purified by reverse phase prep-HPLC on a SpectraSystem P2000 using a 250mm x 21.2 mm Gemini-NX 10µ C18 column eluting a gradient of 40% MeCN in 0.1% formic acid increasing to 70% MeCN over 45 min at a flow rate of 12 ml/min to give BCSR **2** (as a 1:1 mixture of diastereomers) as a deep red solid (16 mg, 18%). Variable trace quantities (≤ 8%) of monocaged sulforhodamine were present in different batches. NMR data for the pure 1:1 mixture of diastereomers A and B: δ_H (500 MHz:$CDCl_3$): 1.34-1.37 ($12H^{A\ and\ B}$, m, 4 × CH_3^A + 4 × CH_3^B), 3.61-3.68 ($8H^{A\ and\ B}$, m, 4 × CH_2^A + 4 × CH_2^B), 6.76 ($1H^{A\ or\ B}$, d, J = 9.1 Hz, Ar-$H^{A\ or\ B}$), 6.79 ($1H^{A\ or\ B}$, d, J = 9.1 Hz, Ar-$H^{A\ or\ B}$), 6.84-6.95 ($5H^{A\ and\ B}$, m, C*H*CF_3^A + C*H*CF_3^B + 4 × Ar-H^A + 4 × Ar-H^B), 7.09 ($1H^{A\ and\ B}$, broad d, J = 9.1 Hz, Ar-H^A + Ar-H^B), 7.13 ($1H^{A\ or\ B}$, q, J = 5.3 Hz, C*H*$CF_3^{A\ or}$

^{B}), 7.16 (1$H^{A\ or\ B}$, q, J = 5.3 Hz, C*H*$CF_3^{A\ or\ B}$), 7.60 (1$H^{A\ and\ B}$, broad t, J = 6.9 Hz, Ar-H^A + Ar-H^B), 7.68 (1$H^{A\ and\ B}$, broad t, J = 6.9 Hz, Ar-H^A + Ar-H^B), 7.78 (1$H^{A\ and\ B}$, broad t, J = 7.5 Hz, Ar-H^A + Ar-H^B), 7.84 (1$H^{A\ and\ B}$, broad t, J = 7.9 Hz, Ar-H^A + Ar-H^B), 7.90-7.98 (2$H^{A\ and\ B}$, m, Ar-H^A + Ar-H^B), 8.11 (1$H^{A\ and\ B}$, d, J = 8.3 Hz, Ar-H^A + Ar-H^B), 8.12 (1$H^{A\ and\ B}$, broad d, J = 7.9 Hz, Ar-H^A +Ar-H^B), 8.18 (1$H^{A\ and\ B}$, d, J = 8.2 Hz, Ar-H^A + Ar-H^B), 8.52 (1$H^{A\ or\ B}$, broad d, J = 7.6 Hz, Ar-$H^{A\ or\ B}$), 8.55 (1$H^{A\ or\ B}$, s, Ar-$H^{A\ or\ B}$), 8.57 (1$H^{A\ and\ B}$, s, Ar-$H^{A\ or\ B}$). δ_C (125 MHz: $CDCl_3$): 12.83 (CH_3), 46.51 (CH_2), 46.60 (CH_2), 72.77 (q, J = 35.1 Hz, $CHCF_3^{A\ or\ B}$), 72.91 (q, J = 35.1 Hz, $CHCF_3^{A\ or\ B}$), 96.89 (CH), 113.53 (CH), 113.56 (CH), 113.79 (C), 114.92 (C), 114.93 (C), 115.57 (CH), 115.61 (CH), 121.65 (q, J = 282.3 Hz, CF_3), 121.87 (q, J = 282.0 Hz, CF_3), 124.25 (C), 124.28 (C), 124.34 (C), 124.37 (C), 125.79 (CH), 125.82 (CH), 125.87 (CH), 129.00 (CH), 129.10 (CH), 129.67 (CH), 130.08 (CH), 130.74 (CH), 130.79 (CH), 130.81 (CH), 132.20 (CH), 132.24 (CH), 132.29 (CH), 132.36 (CH), 134.36 (CH), 134.75 (CH), 134.79 (CH), 134.89 (CH), 134.96 (CH), 135.07 (CH), 135.10 (CH), 137.19 (C), 137.20 (C), 138.11 (C), 138.13 (C), 138.64 (C), 138.69 (C), 147.59 (C), 147.63 (C), 147.85 (C), 147.90 (C), 150.08 (C), 150.09 (C), 155.84 (C), 156.17 (C), 156.19 (C), 157.54 (C), 157.55 (C), 157.67 (C), 157.68 (C). δ_F (470 MHz: $CDCl_3$): -74.93 (d, J = 5.3 Hz), -74.95 (d, J = 5.3 Hz), -75.21 (d, J = 5.3 Hz), -75.22 (d, J = 5.3 Hz). m/z (ESI): Found: 965.1929. $C_{43}H_{39}F_6O_{11}N_4S_2$ requires M^+, 965.1955.

Synthesis of Monocaged Sulforhodamine 3

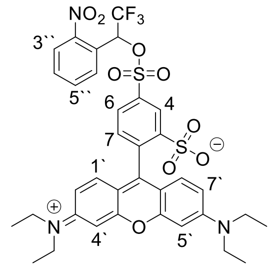

Diisopropylethylamine (90 µl, 0.52 mmol, 3.0 eq) was added to a solution of sulforhodamine B chloride (100 mg, 0.173 mmol), 1-(o-nitrophenyl)-2,2,2-trifluoroethanol (115 mg, 0.52 mmol, 3.0 eq) and DMAP (2 mg, 0.017 mmol, 0.1 eq) in dichloromethane (2 ml). The solution was stirred at RT for three hours, diluted with dichloromethane (10 ml) and washed with 1M hydrochloric acid. The organic layer was dried over magnesium sulfate and concentrated under vacuum. The residue was purified by column chromatography on a 10g SNAP ultra cartridge eluting dichloromethane increasing to 10% MeOH to yield the product as a red solid (66 mg, 50%). δ_H (500 MHz: $CDCl_3$): 1.29 (6H, t, J = 7.3 Hz, CH_3), 1.32 (6H, t, J = 7.3 Hz, CH_3), 3.51-3.61 (8H, m, 4 x CH_2), 6.66 (1H, d, J = 2.3 Hz, H-4` or 5`), 6.67 (1H, d, J = 2.3 Hz, H-4` or 5`), 6.78 (1H, dd, J = 9.5, 2.3 Hz, H-2` or 7`), 6.79 (1H, dd, J = 9.5, 2.2 Hz, H-2` or 7`), 7.05 (1H, d, J = 9.5 Hz, H-1` or 8`), 7.12 (1H, q, J = 5.7 Hz, $CHCF_3$), 7.15 (1H, d, J = 9.5 Hz, H-1` or 8`), 7.21 (1H, d, J = 8.0 Hz, H-7), 7.66 (1H, ddd, J = 8.8, 7.4, 1.5 Hz, H-4``), 7.86 (1H, td, $J$ = 7.7, 1.3 Hz, H-5``), 7.95 (1H, broad d, J = 7.9 Hz, H-6``), 7.98 (1H, dd, $J$ = 8.0, 2.0 Hz, H-6), 8.12 (1H, dd, $J$ = 8.3, 1.3 Hz, H-3``), 8.84 (1H, d, J = 1.9 Hz, H-4). δ_C (125 MHz: $CDCl_3$): 12.73 (CH_3), 46.02 (CH_2), 72.10 (q, J = 35.0 Hz, $CHCF_3$), 95.86 (CH), 113.57 (CH), 113.74 (CH), 114.18 (C), 114.29 (C), 122.07 (q, J = 282.1 Hz, CF_3), 125.30 (C), 125.46 (CH), 127.70 (CH), 129.07 (CH), 130.09 (CH), 130.36 (CH), 131.63 (CH), 133.29 (CH), 133.37 (CH), 134.96 (CH), 136.23 (CH), 136.72 (C), 147.74 (C), 149.44 (C), 155.69 (C), 155.72 (C), 158.02 (C), 158.03 (C), 158.06 (C).δ_F (470 MHz: $CDCl_3$): -75.29 (d, J = 5.7 Hz).m/z (ESI): Found: 784.1586. $C_{35}H_{34}F_3O_9N_3S_2Na$ requires (M^++*Na*), 784.1581. Note diastereotopicity is evident in xanthene unit due to restricted rotation.

General procedure for photouncaging

In a standard cuvette, a stirred solution of caged compound (10 μM) in the desired solvent was irradiated at either 365 nm using a Prizmatix Mic-LED 365 fitted with a cuvette holder at full power (650 mW) or at 595 nm using a Prizmatix FC-5 collimated light fitted with a 595 ± 5 nm bandpass filter (Edmund Optics) on full power (28 mW), again fitted with a cuvette holder. Beam diameter is 2.5 cm in each case. Solutions were irradiated for the desired length of time then analysed by LCMS using a Phenomenex Kinetix 5μ XB-C18 50×4.6 mm column on a Shimadzu LC-2010 LCMS eluting 10-90% (0.1% Formic acid:MeCN) over 20 min. Flow rate 1.0 mL/min. Fluorescence spectra were obtained on a Shimadzu RF-5301PC instrument.

Cell culture and imaging conditions

To assess the localization and uncaging of BCSR, HEK 293T (Human embryonic kidney cell line; American Type Culture Collection; ATCC-CRL-3216) or HeLa cells (Human cervix epitheloid carcinoma cell line; European Collection of Animal Cell Cultures; 93021013) were loaded with 100 nM MitoTracker Green FM (ThermoFisher Scientific) and either 1 μM Tetramethylrhodamine ethyl ester (TMRE) or 1 μM BCSR, or 1 μM BCSR alone. Cells were grown in DMEM (10% FBS and 1% Glutamax, Invitrogen). HEK 293T cells were seeded at 25% confluency in glass bottomed chamber slides (Lab-Tek II, Nunc, ThermoFisher) and allowed to adhere overnight. HeLa cells were seeded at 75,000 cells on 35 mm diameter glass bottom dish (Nunc, ThermoFisher Scientific) and allowed to adhere overnight. Cells were then incubated with 100 nM MitoTracker Green FM (ThermoFisher Scientific) and either 1 μM Tetramethylrhodamine ethyl ester (TMRE) or 1 μM BCSR for 10 min in the dark and washed 3 times with DMEM. HEK 293T cells were imaged at room temperature using an inverted epi-fluorescence microscope (TE2000U, Nikon) equipped with a 100X objective lens, and an iXon EMCCD camera (Andor). Widefield excitation (488 nm or 555 nm) was provided by a monochromator (DeltaRAM, PTI), and targeted ultraviolet irradiation by a computer-controlled photolysis laser (~2 μm diameter; Rapp Optoelektronic). Images were acquired using uManager software and analysed using FIJI software. HeLa cells were imaged at 37°C in a CO_2 controlled chamber (Oko Lab) using a spinning disc microscope (ANDOR Dragonfly 200) and a 100x objective lens (Nikon). HeLa cells were further incubated in 10 μM FCCP (Sigma Aldrich) for 10 min, or illuminated by transmitted light (400-800 nm) for 5 min followed by digitonin (Sigma Aldrich) addition (final concentration of 25 μg/ml) for another 5 mins. 488 and 561 nm lasers were used for excitation. Images were captured using a iXon EMCCD camera and Fusion software (ANDOR) and analysed using the Imaris software (Bitplane). To assess the colocalization, a complete z-stack of the cell was acquired. Z-stacks were processed using Imaris (Bitplane) to produce a 3D maximum projection image.

To assess the localistion of monocaged sulforhodamine **3,** HeLa cells were seeded at 75,000 cells on 35 mm diameter glass bottom dish (Nunc, ThermoFisher Scientific) and allowed to adhere overnight. The medium was removed by aspiration and replaced with DMEM, 10% FBS and 1% Glutamax (Invitrogen). Cells were then incubated with 300 nM DAPI (ThermoFisher Scientific) for 5 min in the dark and washed 3 times with DMEM. The cells were subsequently incubated with 1 μM MCSR for 10 min in the dark and washed 3 times with DMEM. Cells were imaged before and after MCSR addition using Zeiss LSM880 confocal system equipped with Zeiss Plan-Achromat 63x/1.4 NA oil immersion objective. 360 and 561 nm lasers were used for excitation of DAPI and MCSR, respectively. Z-stacks were acquired at 0.5-μm steps and processed using Imaris (Bitplane) to produce a 3D maximum projection image.

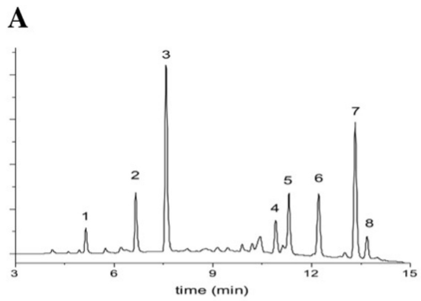

Peak	m/z	Assignment
1	503	Sulforhodamine minus 2 x Ethyl
2	531	Sulforhodamine minus Ethyl
3	559	Sulforhodamine
4	706	Mono Caged sulforhodamine minus 2 x Ethyl
5	762	Compound 4
6	734	Mono Caged sulforhodamine minus Ethyl
7	762	Compound 3
8	965	Compound 2

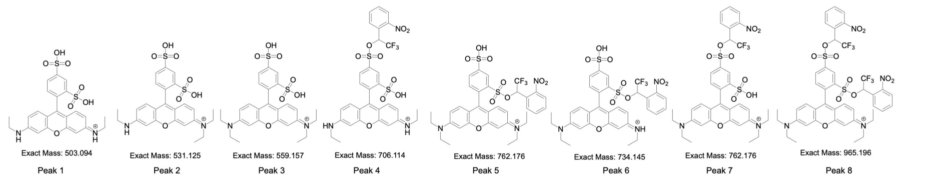

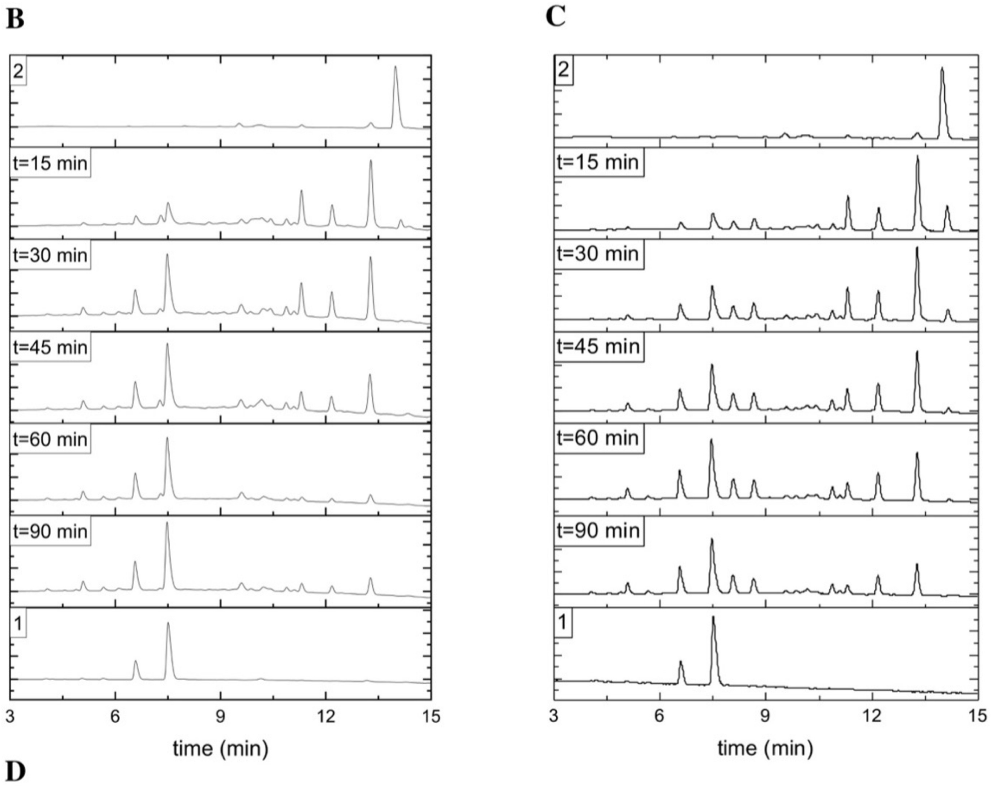

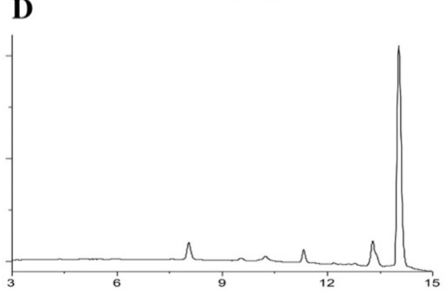

Figure S1 (A) LCMS-UV (254 nm) trace of uncaging of BCSR **2** after 30 min irradiation at 365 nm (10 µM in pH 7.4 buffer) and assignment of peaks and structures based on m/z data. LC-UV (254 nm) after the irradiation of BCSR **2** at 365 nm light (B) or 595 nm (C) over time (10 µM in pH 7.4 buffer) in comparison with a 10 µM solution of sulforhodamine B **1** in pH 7.4 buffer (commercially available sulforhodamine B (CAS: 3520-41-1) is only ~75% pure with the remainder being the dye minus an ethyl group). (D) LC-UV (254 nm) trace showing stability of BCSR **2** after 4 days at 37 °C in pH 7.4 buffer (10 µM).

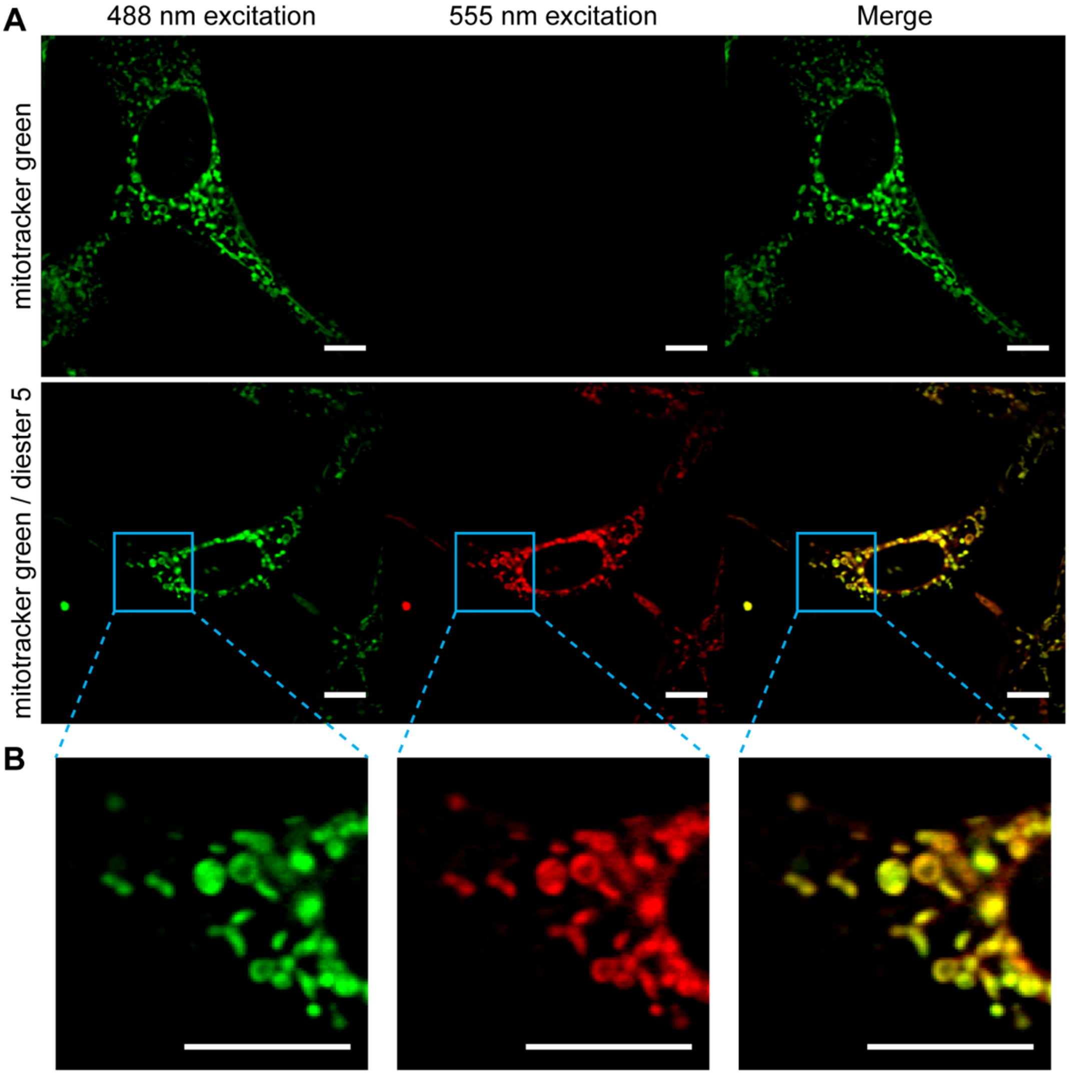

Figure S2 BCSR **2** colocalises with mitochondria in HEK293T cells. (A) Representative images showing the fluorescence signal obtained with 488 nm excitation (left) and with 555 nM excitation (middle), and a composite merge of the two channels (right). (A) upper panel, when HEK293T cells were incubated with mitotracker green (100 nM) alone (top), punctate mitochondrial staining was apparent when the cells were excited with 488 nm excitation. No signal was detected in these same cells when illuminated with 555 nm excitation light. (A) lower panel, when HEK293T cells were loaded with mitotracker green (100 nM), to show mitochondria position, and caged TFNB sulfonate diester 5 (diester 5), there was substantial overlap in the localisation of the fluorescence from both compounds. As indicated by composite colour images (right), the signal observed as a result of diester 5 loading originated from the same subcellular component as that of mitotracker green. (B) Magnified regions of cells (blue box) indicated in A. All scale bars = 10 µm.

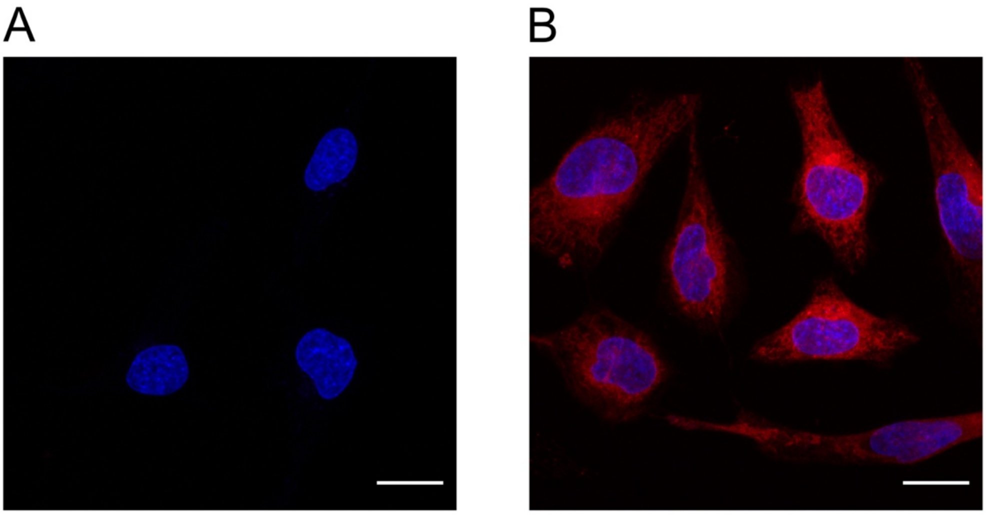

Figure S3 Cell uptake and localisation of monocaged sulforhodamine **3**. HeLa cells were incubated with 300 nM DAPI (Sigma Aldrich) for 5 min before exposure to 1 μM monocaged sulforhodamine 3 for 10 min in the dark and washed 3 times with DMEM. (A) Representative image after DAPI incubation and before exposure to monocaged sulforhodamine 3. (B) Representative image after the 10 min of exposure to monocaged sulforhodamine 3. Both images were excited with 360 nm (DAPI) and 561 nm (monocaged sulforhodamine) lasers.

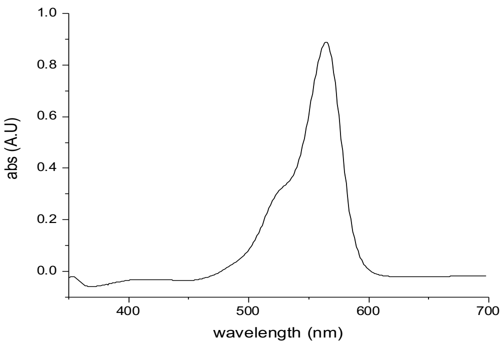

Figure S4 UV/Vis absorption spectra of BCSR **2** (10μM in pH7.4 buffer)

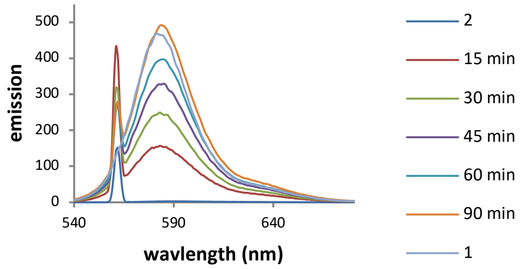

Figure S5 Fluorescence increase after the irradiation of BCSR **2** at 595 nm light over time (10 μM in pH 7.4 buffer) in comparison with a 10 μM solution of sulforhodamine B **1** in pH 7.4 buffer (excitation 560 nm)

Video Legends

Video 1. BSCR 2 uncaging in a single HEK 293T cell. Five-minute recording showing a single HEK 293T cells loaded with diester 2 (100 nM), visualized using a 100x objective lens. Diester 2 is uncaged upon excitation with 555 nm light, causing an increase in the fluorescence signal. Data also shown in Figure 4. Scale bar = 10 µm.

Video 2. BSCR 2 uncaging in a field of HEK 293T cells. Three-minute recording show a field of HEK 293T cells loaded with diester 2 (100 nM), visualized using a 100x objective lens. Upon excitation with 555 nm light, diester 2 is uncaged in cells throughout the field of view. Scale bar = 10 µm.

Video 3. BSCR 2 uncages in visible light. Two-minute recording showing a field of HEK 293T cells loaded with diester 2 (100 nM), visualized using a 40x objective lens. Diester 2 is uncaged upon excitation with 555 nm light, causing an increase in the fluorescence signal. Scale bar = 40 µm.

Video 4. Selective uncaging of BSCR 2. Video showing that diester 2 can be selectively uncaged by targeting the excitation light to specific cells. In this video, the initial field of view shows cells in which BSCR 2 has already been uncaged (see Video 1). Cells out with the initial field of view have not previously been imaged. In these cells, diester 2 has not been uncaged, are the fluorescence signal is lower. Scale bar = 40 µm.

^{1}H, ^{13}C and ^{19}F NMR spectra of BCSR **2**.

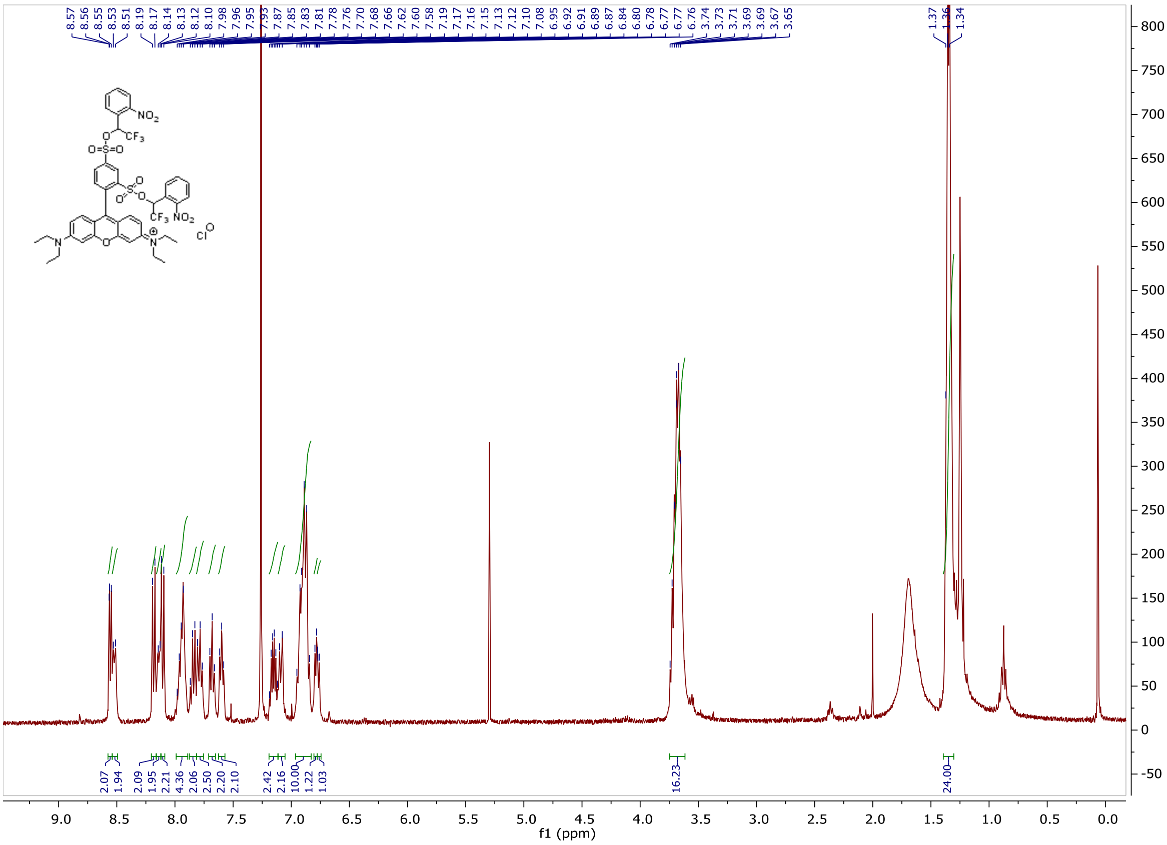

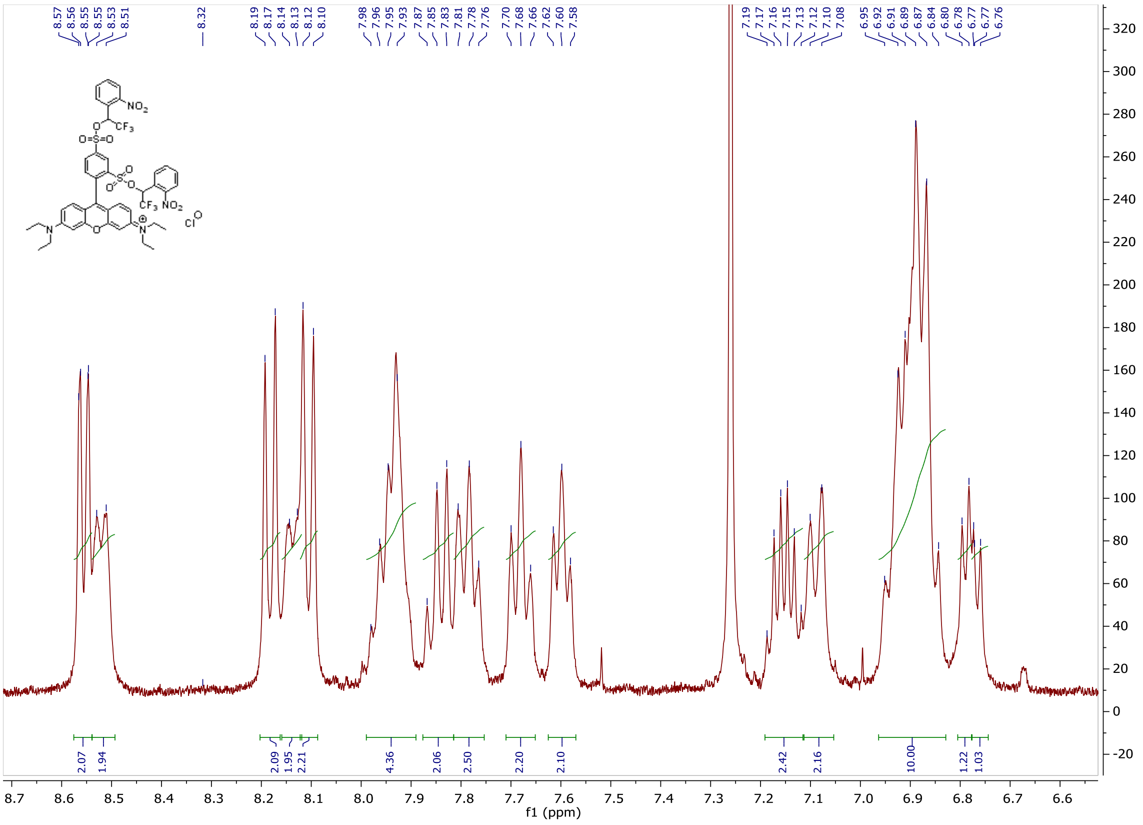

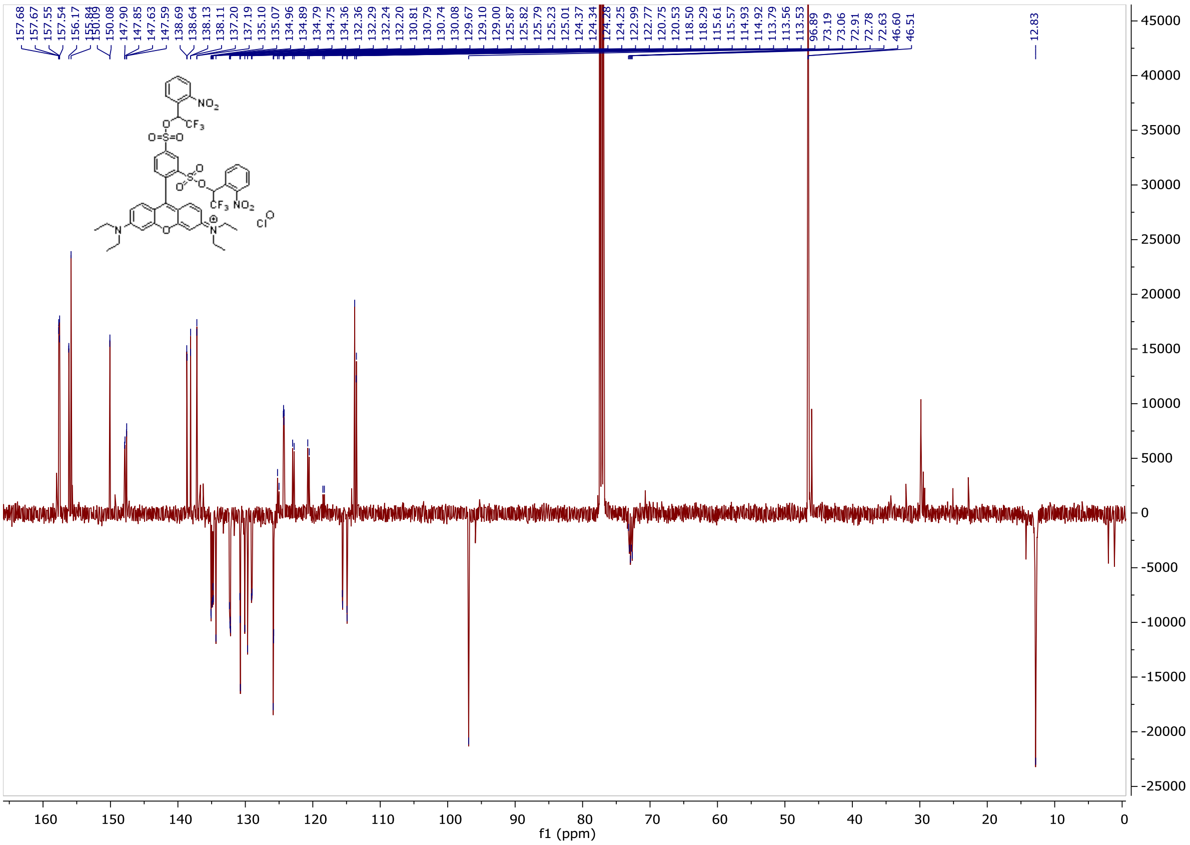
157.68
157.67
157.55
157.54
156.17
150.08
147.90
147.85
147.63
147.59
138.69
138.64
138.13
138.11
137.20
137.19
135.10
135.07
134.96
134.89
134.79
134.75
134.36
132.36
132.29
132.24
132.20
130.81
130.79
130.74
130.08
129.67
129.10
129.00
125.87
125.82
125.79
125.23
125.01
124.37
124.28
124.25
122.99
122.77
120.75
120.53
118.50
118.29
115.61
115.57
114.93
114.92
113.79
113.56
113.53
96.89
73.19
73.06
72.91
72.78
72.63
46.60
46.51
12.83
NO2
CF3
O=S=O
Cl
f1 (ppm)

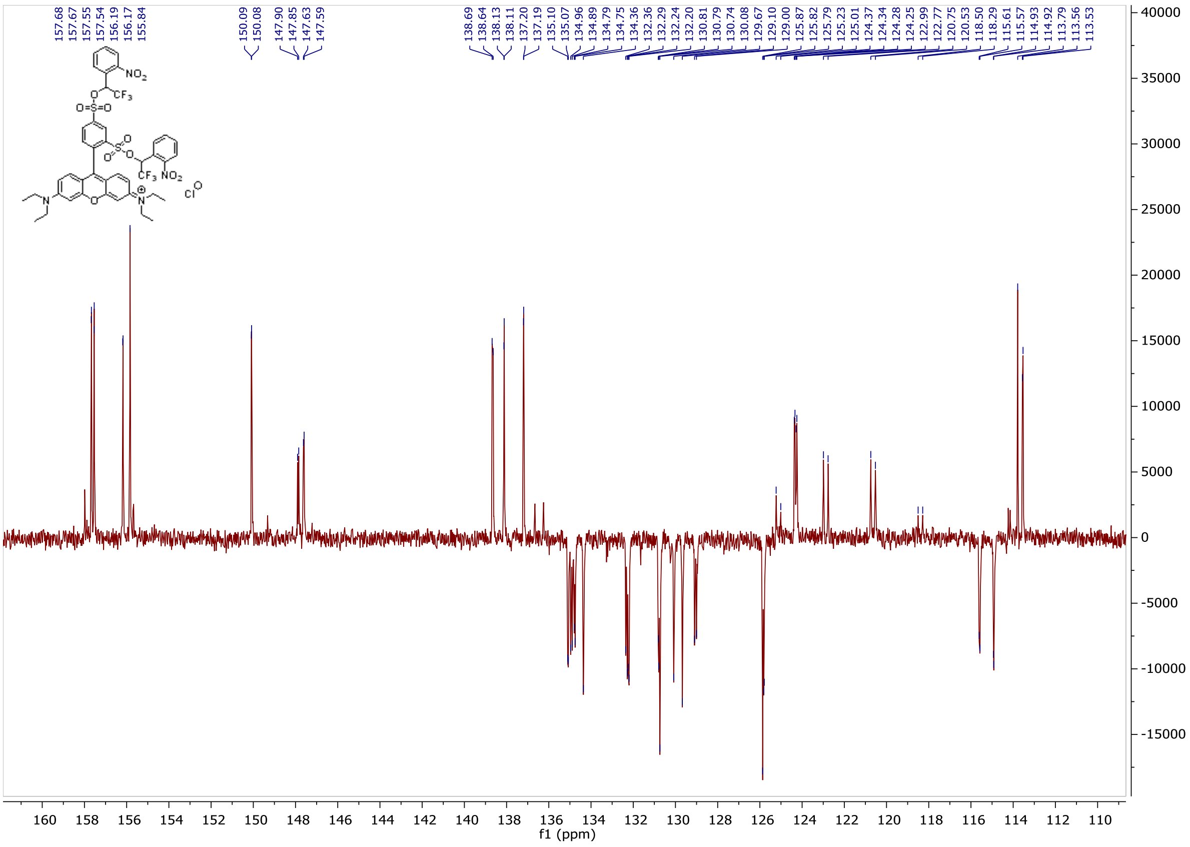
157.68
157.67
157.55
157.54
156.19
156.17
155.84
150.09
150.08
147.90
147.85
147.63
147.59
138.69
138.64
138.13
138.11
137.20
137.19
135.10
135.07
134.96
134.89
134.79
134.75
134.36
132.36
132.29
132.24
132.20
130.81
130.79
130.74
130.08
129.67
129.10
129.00
125.87
125.82
125.79
125.23
125.01
124.37
124.34
124.28
124.25
122.99
122.77
120.75
120.53
118.50
118.29
115.61
115.57
114.93
114.92
113.79
113.56
113.53
NO2
CF3
O=S=O
Cl
f1 (ppm)

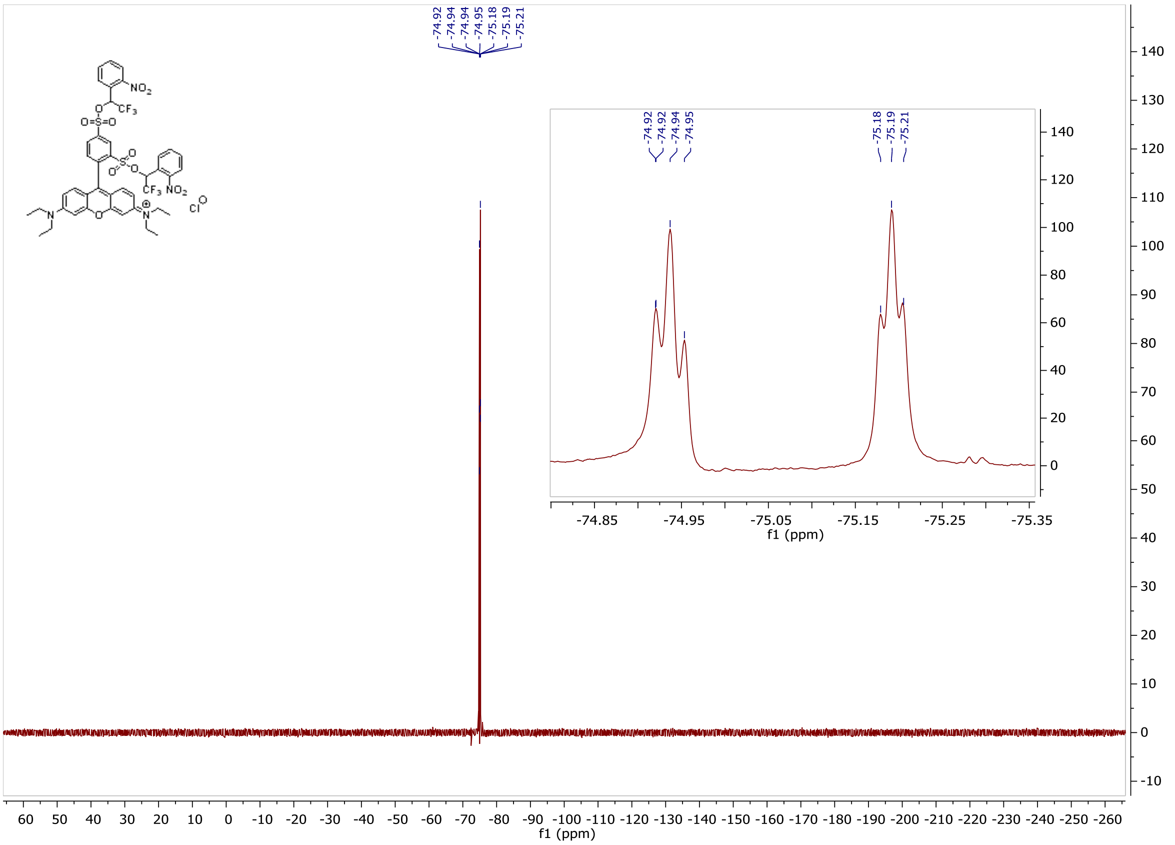
-74.92
-74.94
-74.94
-74.95
-75.18
-75.19
-75.21
NO2
CF3
Cl
-74.85
-74.95
-75.05
-75.15
-75.25
-75.35
f1 (ppm)

^{1}H, ^{13}C and ^{19}F NMR spectra of **3**.

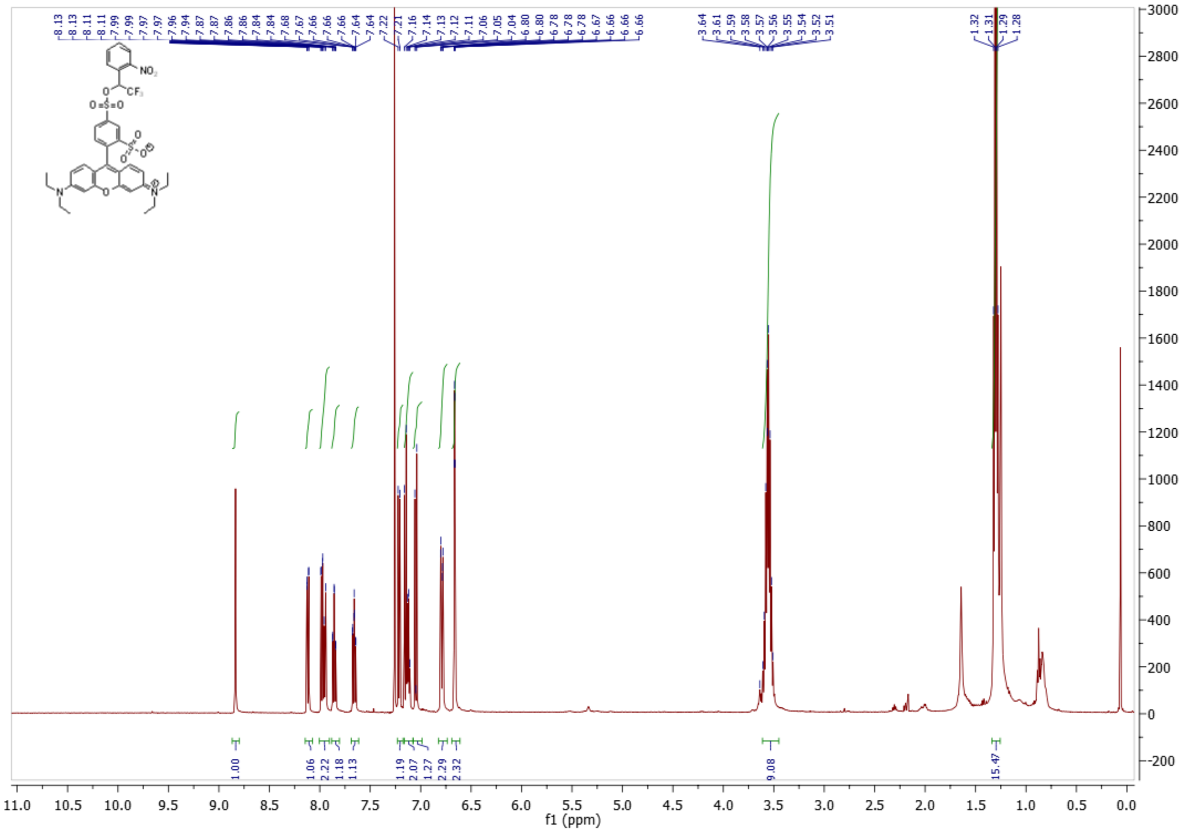

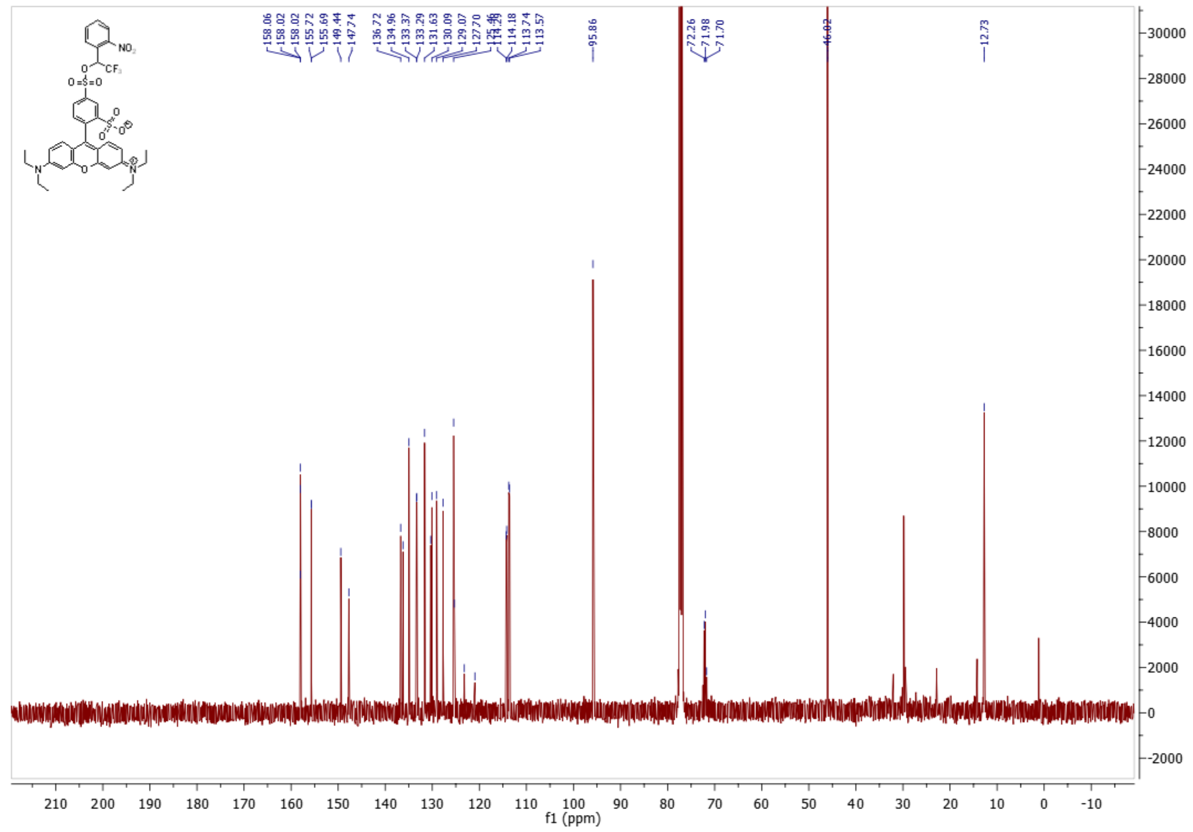

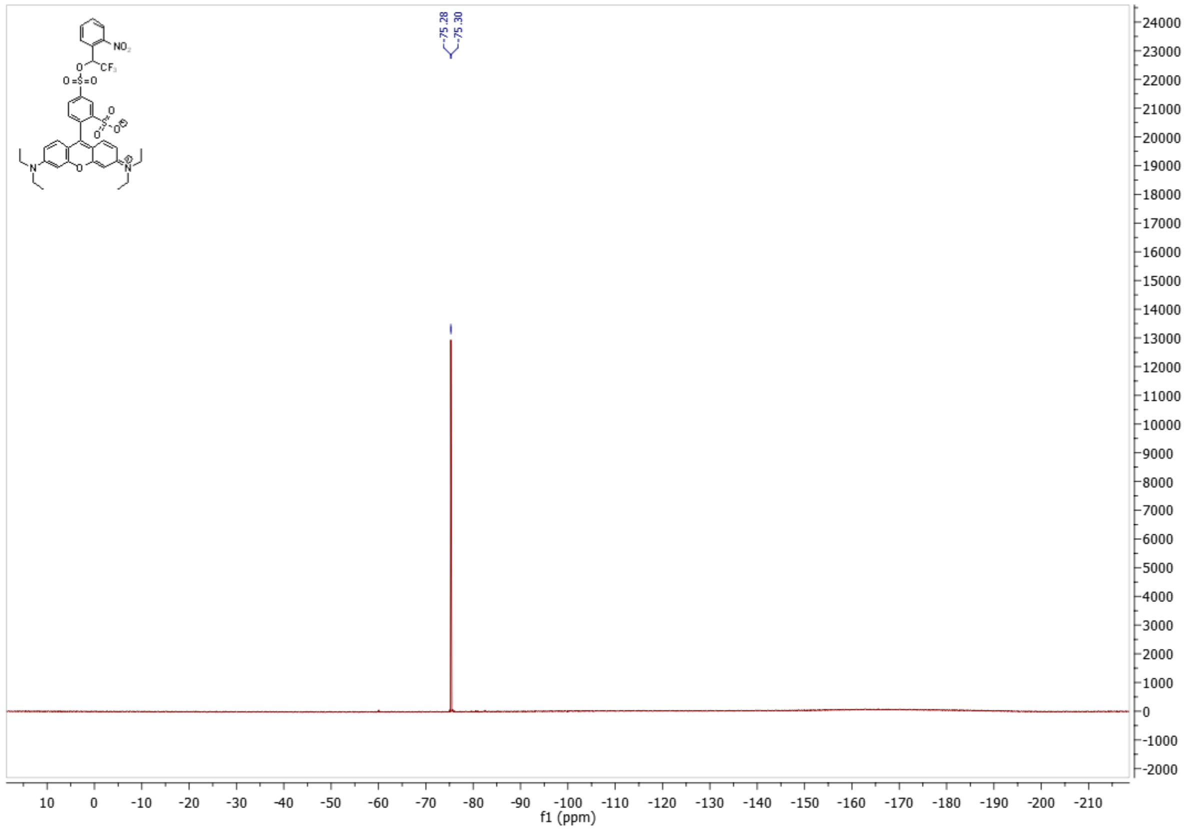

NO2
CF3
-75.28
-75.30
f1 (ppm)
